# A Wonderful Piece of Mechanism

**Published:** 1885-11

**Authors:** 


### A WONDERFUL PIECE OF MECHANISM.

In the August number of this Journal, page 451, we gave an account of an extirpation of the larynx, by Prof. Roswell Park, of the University of Buffalo. The operation was one of the most formidable that is known to surgery. This was, we believe, the third time in which it had been performed in this country. In the first case the patient died in about seven hours. The other lived eight months, but never made a full recovery. The operation was original with Billroth, but it has since been attempted a number of times in Europe.

Dr. Park's operation was performed upon a man of sixty-four years of age, a physician, who was suffering from epithelioma of the larynx, on June 28th. October 3d he was brought before the members of the Faculty and the class in Surgery, and the exhibition was certainly an astonishing one. He seemed in perfect health, and talked, breathed and ate, using his mouth like an ordinary individual, although all the larynx, a portion of the tracheal tube, and the uvula had been removed. These functions were performed by means of a very ingenious artificial larynx, the invention of a pupil of Billroth. It consists of two silver tubes, locking together at the centre, which when joined present this general contour: —^— Each arm of this improvised illustration may represent one of the tubes, and at the central part, which is opposite the opening in the trachea, an oral plate may be imagined. One tube is thrust up into the opening of the pharynx, and the other down into the trachea. When an external opening in the circular plate is closed by a stopper, which is under the control of the hand of the patient, he breathes through the tubes and pharynx. When this is opened he breathes through the tracheal opening. When he eats, the artificial larynx is in this latter condition. The tubes being closed, the food cannot pass down the trachea, but must slip down the esophagus, thus obviating the necessity for an epiglottis. There is also an artificial esophageal opening, and he may be fed through this if for any reason he does not choose to place the food in his mouth and use the artificial larynx.

When he desires to talk, the stopper at the centre of the apparatus is removed, and a piece of mechanism containing a reed like that of a melodeon is inserted. The air from the lungs passing over this causes vibrations, which are modulated by the oral muscles. It is, in fact, an artificial vocal cord, and with it the speech is as distinct and as perfectly articulate as that of the normal vocal organ. Of course it is entirely a monotone, for there is but one vibrating organ, and there can be no change of pitch in the tone. But the same is the case with the ordinary jewsharp, to which the action of this may be compared, yet all the effects of a change of pitch are simulated by the use of the oral muscles, especially the buccinators. Certainly, the articulation of the subject of this operation is very surprising, and as a musical instrument the single reed of the apparatus is capable of effects to which those of the one violin string of Paganini bore no comparison. By using the appliance without the reed the patient is enabled to whisper very loudly, for in whispering the vocal cords take no part.

After the operation the man was fed for three weeks by means of a rubber tube, which was thrust through the wound, down the esophagus into the stomach, but since that time he has been able to take food through the mouth. All the changes in the apparatus are made at the external opening, and the whole is removable at pleasure. Unless the chin be raised nothing unusual is observable, for the oval plate, less than an inch in diameter, is hidden by a high collar. The patient is a great lover of tobacco, and one of his principal gratifications is that he is now able to chew and smoke at pleasure, without danger of a recurrence of the epithelioma—at least in the larynx.

